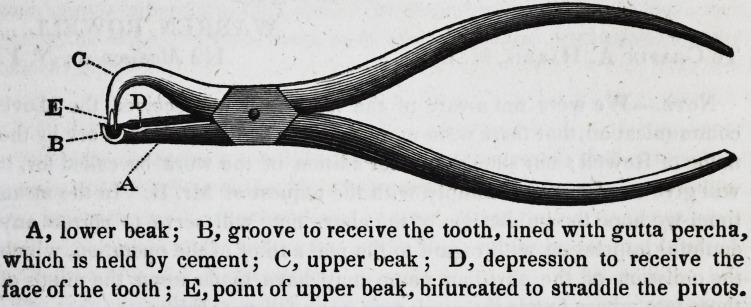# Letter from Samuel Rambo

**Published:** 1850-10

**Authors:** Samuel Rambo


MONTGOMERY, ALA, *July* 29*th*, 1850.

DR. C. A. HARRIS:

*Dear Sir:*—I herewith send you a sketch of an instrument, the invention of which is claimed by J. W. Torrence, a dentist of Loundsboro, Ala. From *experience*, I must say, it is the best for holding teeth, for the purpose of grinding them, that I have seen. It is at your service, and of the profession generally.

Yours, very respectfully,
SAMUEL RAMBO.

A, lower beak; B, groove to receive the tooth, lined with gutta percha, which is held by cement; C, upper beak; D, depression to receive the face of the tooth; E, point of upper beak, bifurcated to straddle the pivots.

## Figures and Tables

**Figure f1:**